# Rethinking Care: Early Palliative Support for Advanced Heart Failure

**DOI:** 10.7759/cureus.82106

**Published:** 2025-04-11

**Authors:** Argavan Ansari, Vijay Sookai, Isabel Gossen, Samy Matta, Hassan Mahmudul, Roxana Lazarescu

**Affiliations:** 1 Internal Medicine, Touro College of Osteopathic Medicine, New York, USA; 2 Medicine and Surgery, Touro College of Osteopathic Medicine, New York, USA; 3 Internal Medicine, Wyckoff Heights Medical Center, Brooklyn, USA; 4 Internal Medicine, Alexandria University, Alexandria, EGY; 5 Internal Medicine, St. George's University School of Medicine, St. George's, GRD

**Keywords:** advanced disease, caregiver burden, congestive heart failure, end-of-life care, healthcare access, hospice transition, palliative care, quality of life, symptom management, systemic barriers

## Abstract

Congestive heart failure (CHF) remains a major global cause of mortality, impacting millions worldwide. It is characterized by the heart’s inability to pump adequate blood, leading to progressive symptoms such as dyspnea and fluid retention. This case report highlights an 87-year-old male with complex multi-system disease, including heart failure with reduced ejection fraction, chronic obstructive pulmonary disease (COPD), diabetes, chronic kidney disease, atrial fibrillation, prior intracranial hemorrhage, and multiple malignancies. Despite numerous hospitalizations for acute hypoxic respiratory failure, exacerbated by CHF, COPD, pleural effusions, and suspected hospital-acquired pneumonia, his condition continued to deteriorate. Standard treatments, including bilevel positive airway pressure, diuresis, bronchodilators, and antibiotics, provided only temporary relief, and cognitive and functional status declined.

A key challenge in his care was his daughter, his sole caregiver, who faced significant emotional and financial strain. She did meet the criteria for caregiver burden. Misconceptions about hospice care contributed to her reluctance to transition away from aggressive treatment, delaying access to early palliative care and symptom management. Systemic barriers, such as gaps in insurance coverage, further compounded caregiving difficulties by limiting access to home health services and necessary support.

This case underscores the critical need for early integration of palliative care in managing advanced CHF. Early involvement can improve symptom control, enhance patient comfort, and provide vital support for caregivers. Proactive discussions around the limitations of aggressive treatment, along with efforts to dispel common misconceptions about hospice care, can help reduce unnecessary hospitalizations and ensure that care remains aligned with the patient's values and goals.

In applying the Institute for Healthcare Improvement's (IHI) 5Ms (medications, mentation, mobility, multi-complexity, and what matters most) model, providers are reminded to take a comprehensive, person-centered approach to geriatric care. Each domain plays a role in addressing the complex needs of older adults, but above all, focusing on what matters most helps ensure that care decisions reflect the individual’s preferences, priorities, and definition of quality of life. Aligning treatment with these personal goals promotes dignity, reduces burdensome interventions, and supports more meaningful, compassionate care throughout the disease trajectory.

## Introduction

It can be challenging when it comes to managing patients with coexisting multi-system diseases. Especially when a patient's health succumbs to these advanced illnesses. Whether it is cancer, dementia, or heart disease, a question then emerges on how to provide the optimal care to those end-of-life patients. But the decision on how to provide this care is then complicated by different cultural, religious, social, and ethical practices and financial drawbacks. According to the World Health Organization, each year about 56.8 million people, including 25.7 million in the last year of life, need palliative care, and only about 14% of people who need palliative care currently receive it [1]. These numbers are the result of different barriers, including the ones mentioned above. Here, we present a case of an 87-year-old patient with congestive heart failure (CHF) accompanied by other advanced illnesses, who required palliative care, but his access to proper care was hindered.

## Case presentation

This case involves an 87-year-old male with a complex and advanced medical history, including heart failure with reduced ejection fraction (20-25%), chronic obstructive pulmonary disease (COPD) requiring 5L home oxygen, type 2 diabetes mellitus, stage 3 chronic kidney disease (CKD), atrial fibrillation, prior intracranial hemorrhage, and multiple malignancies. He was admitted multiple times in recent months for acute hypoxic respiratory failure. His most recent episode was likely due to concurrent exacerbations of COPD and heart failure, complicated by bilateral pleural effusions and suspected hospital-acquired pneumonia. Despite multiple medical interventions, including bilevel positive airway pressure, diuresis, bronchodilators, and antibiotics, his clinical trajectory continued to decline. He experienced a steady worsening of both functional and cognitive status over time.

The patient’s care was further complicated by psychosocial factors, particularly the involvement of his daughter, who was his sole caregiver and surrogate decision-maker. She lived with the patient and provided full-time care. She faced significant emotional and financial stress, reporting no financial support despite her father’s desire for her to accept it. She declined external caregiver assistance due to personal, moral, and ethical beliefs, stating that it was her duty to care for her father without compensation.

Despite clear evidence of his poor prognosis and the limitations of continued aggressive treatment, the daughter initially resisted transitioning to hospice care. She expressed concerns that accepting hospice would mean “giving up,” a belief that delayed the patient’s access to appropriate palliative services. These hesitations were explored by the care team during late-stage discussions.

Palliative care was consulted late in the course of hospitalization, at which point the patient had already experienced multiple hospitalizations and progressive decline. This delay in consultation limited the opportunity for earlier symptom management, advanced care planning, and caregiver support. The daughter ultimately came to understand the goals of hospice care following multiple discussions with the palliative care team, who emphasized comfort, safety, and improved support at home. At this point, inpatient hospice was offered to stabilize the patient and better support both him and his daughter.

Diuresis was discontinued during the hospitalization due to worsening kidney function, underscoring the limitations of continuing aggressive interventions in the setting of advanced multi-organ disease. Additionally, gaps in insurance coverage restricted access to home health services and necessary caregiver support, presenting systemic barriers that further complicated care.

This case highlights the ethical and practical complexities of managing a geriatric patient with advanced, coexisting conditions. It emphasizes the importance of early palliative care involvement, timely goals-of-care discussions, and supportive services to reduce caregiver burden and improve patient outcomes. Earlier integration of palliative care could have supported better symptom relief, clarified care goals, and eased the transition to hospice, all while aligning care more closely with the patient’s and family’s values.

## Discussion

Based on the National Health and Nutrition Examination Survey, the prevalence of heart failure (HF) in the US in 2020 was about 6.8 million people (90% CI = 5,870,291-8,343,622), and is expected to increase to 11.4 million people by 2050 [1,2]. HF is estimated to be a contributing cause in 417,539 deaths in the United States in 2023, according to the CDC Wonder Online Database [3]. In 2018, 2.73% of HF patients died during their hospital stay, 6.32% within 30 days of discharge, and 26.95% within one year of discharge [4]. When symptoms are refractory to treatment, and the patient is not a candidate for transplant or mechanical support, what is left is palliative and end-of-life (EOL) care. Therapy goals for HF are to improve quality of life, manage symptoms, decrease hospitalization, and reduce mortality. Medical management and mechanical support via left ventricular assist device (LVAD) or extracorporeal membrane oxygenation (ECMO) for stage D CHF are used to achieve those goals [5]. Absolute contraindications for mechanical support include irreversible renal disease and malignancies, with relative contraindications of age over 80 for destination therapy, severe peripheral vascular disease, and impaired cognitive function [6]. Most heart transplant programs have an absolute contraindication for age over 80 [7]. There are financial considerations to take as well. The cost for an LVAD, including implantation, hospital admissions, and follow-up, was found to be $726,200 in 2016 US dollars over six years [8]. The financial burden is a barrier in itself, but it is seen as one of the only few effective treatments for advanced HF and is essential for acting as a bridge to heart transplant. Our patient has multiple comorbidities that would prevent him from meeting the criteria for both mechanical support and transplant, which makes EOL discussions and palliative care in the best interests of this patient.

The necessity for EOL planning becomes more important as people live longer, estimations of HF prevalence increasing over time, and considering mortality rates after hospitalization. However, barriers exist to accessing EOL planning and palliative care. One barrier is healthcare providers' attitudes and perceptions. A Mayo Clinic Health System survey showed that clinicians were hesitant to discuss EOL care due to their discomfort with the matter, perception of patient and family readiness, fear of destroying hope, and lack of time. In addition, there was disagreement regarding whose responsibility it was to handle these discussions [9]. Evidence indicates that most patients do not report ever having EOL discussions with clinicians, suggesting that there is room for improvement in the quality and how often these discussions occur [10]. These issues were laid bare in this case.

There is also the question of when precisely palliative care and palliative therapies should be initiated for HF patients. It is difficult to do this because treatment is based on prognosis, and clinical presentation varies widely from individual to individual. Some patients can live with HF, while others die unexpectedly. According to the American Heart Association, palliative care and palliative therapies have a role, even in the early stages of HF. Palliative care would have a small role early on and should increase over the course of the disease. Palliative therapy and optimal medical therapy should be about equal if the patient transitions to advanced HF, with palliative care dominating near the end of the disease course [11].

We have also taken the patient's and family's cultural background into account. The patient is from Romania, with their country's palliative care capacity focusing on the hospice model [12]. With the potential for hospice being the only thing associated with palliative care, this contextualizes the family's perception of "giving up" as hospice is reserved for patients immediately close to dying, not focusing on maintaining symptom relief and increasing patient comfort. This is in contrast to concepts of palliative care in Western Europe, where multiple Western European countries demonstrated having adequate palliative care capacity for their populations. Romania was classified as a country with a high palliative care need, with low capacity and a low number of specialized services [13]. Palliative care in the US also needs improvement, as most care is provided in hospitals, resulting in coverage gaps in an outpatient setting [14]. This patient would have benefited from an early palliative care consult, where misconceptions could have been addressed regarding palliative care capabilities and hospice, and potentially provided an avenue for the patient to create advanced directives and make decisions regarding his health with his daughter.

Despite receiving optimal medical treatment, the patient experienced 10 hospital admissions and frequent ED visits over the last six months due to episodes of decompensated HF. His condition severely impacted his quality of life, causing persistent symptoms such as shortness of breath, fatigue, and edema, even at rest. Given his New York Heart Association (NYHA) class IIIB/IV status and the limited effectiveness of ongoing medical interventions, the patient was eligible for hospice.

The patient is an adult with the capacity to make decisions for his healthcare. However, he is dependent and has deferred medical decision-making to his daughter, who is the sole caregiver and family member of this patient. Beyond the physical suffering that the patient endures due to his advanced HF, the patient was worried about his daughter's health and her financial status, as she does not get any financial support to take care of him. The frequent hospital admissions and the debilitating symptoms have left him feeling like a burden on his loved ones. The patient also does not like to spend his valuable time in the hospital. Getting hospice support would be in line with his wishes to get as much help at home as possible and support his daughter. Despite multiple conversations and information provided to the patient and the daughter, she declined any assistance for herself as his primary caregiver. She feels it is her duty as a daughter, and it is against her moral ethics to receive any monetary benefits for the care of her dad. She declines this even if it is her father's wish [15].

## Conclusions

This case highlights the importance of integrating palliative and hospice care into the management of advanced heart failure, as heart failure is more than a clinical diagnosis; it is a chronic, progressive illness that affects every aspect of a patient’s life. A patient-centered approach during the final stages is essential, especially for those who prioritize being at home and maintaining dignity while preserving their connection to family. By aligning treatment goals with the patient’s values and preferences, as this patient clearly expressed through his desire to avoid further hospitalizations and reduce his daughter’s burden, we can meaningfully enhance quality of life, even in the absence of curative options. Managing advanced heart failure requires a holistic framework that extends beyond medical stabilization. In this case, a major factor influencing care was not only the patient’s complex medical condition, but also the social, emotional, and cultural dimensions surrounding his care. His daughter's reluctance to accept help, including hospice or financial assistance, reflected deeply rooted personal and cultural beliefs. These factors contributed to delays in transitioning from aggressive treatment to supportive care and underscore the need for early, ongoing goals-of-care discussions that are sensitive to both the patient’s and family’s background. Importantly, this case also reveals opportunities for improved coordination between the primary care provider (PCP), specialists (such as cardiologists and pulmonologists), and the inpatient care team. Although the patient had frequent healthcare interactions due to repeated hospitalizations, there was no early integration of palliative care, nor was there evidence of advanced care planning prior to this final admission. PCPs are often best positioned to initiate these discussions early in the disease course due to their longitudinal relationship with patients and families. However, in complex cases involving multiple comorbidities, specialists may focus narrowly on disease-specific management, which can unintentionally delay conversations about prognosis and quality of life. Strengthening collaboration between PCPs and specialists, and ensuring clear communication about prognosis, functional decline, and treatment limitations, can facilitate more timely transitions to palliative or hospice care.

In addition, educating providers across disciplines about the benefits of early palliative care integration is crucial. Many clinicians, especially in acute care settings, remain hesitant to initiate end-of-life conversations, often due to discomfort, lack of time, or fear of taking away hope. Enhancing provider training on how to approach these sensitive topics, recognize the signs of functional and prognostic decline, and engage families in compassionate, culturally aware discussions can improve patient outcomes and reduce unnecessary interventions. Provider education should also include a clearer distinction between palliative care and hospice, helping clinicians communicate these options more effectively to families who may have misconceptions. Ultimately, this case demonstrates that improving care for patients with advanced heart failure means creating systems that support not only the patient’s physical needs, but also the emotional, cultural, and caregiving context in which they live. A multidisciplinary approach, led by patient values and supported by well-coordinated teams, can reduce suffering, prevent caregiver burnout, and offer dignity in the final stage of life.
